# Human-associated microbiota suppress invading bacteria even under disruption by antibiotics

**DOI:** 10.1038/s41396-021-00929-7

**Published:** 2021-03-12

**Authors:** Andrew D. Letten, Michael Baumgartner, Katia R. Pfrunder-Cardozo, Jonathan M. Levine, Alex R. Hall

**Affiliations:** 1 School of Biological Sciences, University of Queensland, Brisbane, QLD Australia; 2 Department of Environmental Systems Science, Institute of Integrative Biology, ETH Zürich, Zürich, Switzerland; 3 Dept of Ecology and Evolutionary Biology, Princeton University, Princeton, NJ USA

**Keywords:** Microbial ecology, Community ecology, Antibiotics

## Abstract

In light of their adverse impacts on resident microbial communities, it is widely predicted that broad-spectrum antibiotics can promote the spread of resistance by releasing resistant strains from competition with other strains and species. We investigated the competitive suppression of a resistant strain of *Escherichia coli* inoculated into human-associated communities in the presence and absence of the broad and narrow spectrum antibiotics rifampicin and polymyxin B, respectively. We found strong evidence of community-level suppression of the resistant strain in the absence of antibiotics and, despite large changes in community composition and abundance following rifampicin exposure, suppression of the invading resistant strain was maintained in both antibiotic treatments. Instead, the strength of competitive suppression was more strongly associated with the source community (stool sample from individual human donor). This suggests microbiome composition strongly influences the competitive suppression of antibiotic-resistant strains, but at least some antibiotic-associated disruption can be tolerated before competitive release is observed. A deeper understanding of this association will aid the development of ecologically-aware strategies for managing antibiotic resistance.

The overuse of broad-spectrum antibiotics in clinical and agricultural settings is a key driver of the current antibiotic resistance crisis [1]. Research into antibiotic resistance has traditionally focused on the evolution of resistance in individual pathogens [2]. In the last decade, researchers have turned their attention to the collateral damage inflicted on commensal members of the microbiome, such as those belonging to the dense communities of the human gastrointestinal tract [3, 4]. Several studies have shown that antibiotics can leave gut communities vulnerable to colonisation by other pathogens [5–7], and, most recently, resistance evolution in invading strains can be facilitated by the absence of community suppression [8, 9]. Taken together, these two lines of enquiry appear to bear out conventional wisdom that relative to narrow-spectrum antibiotics or antibiotic-free conditions, broad spectrum antibiotics should increase the likelihood of communities being invaded by resistant strains [10, 11]. On the other hand, given evidence that community-level properties can sometimes be robust to changes in taxonomic composition [12], it is possible that some antibiotic-associated disruption can be tolerated before colonization resistance is affected. Despite the importance of these contrasting predictions, there have been few, if any, direct tests in human-associated microbiota.

We investigated the effect of broad and narrow spectrum antibiotics on the strength of competitive suppression on a resistant variant (generated by in vitro selection for resistance mutations) of a focal strain (*Escherichia coli* K-12 MG1655) inoculated into gut microbiome communities collected from human faecal samples. The focal strain was jointly resistant to the broad-spectrum antibiotic rifampicin (targets Gram-positives and Gram-negatives via inhibition of the highly conserved bacterial RNA polymerase) and the narrow spectrum antibiotic polymyxin B (only targets Gram-negatives). The focal strain was inoculated alongside live or sterile slurry produced using a sample from one of three healthy human donors (described in [9]) into customized gut media without antibiotics or supplemented with 128 *μ*g/ml rifampicin or 4 *μ*g/ml polymyxin B (see Fig S1). Following 24 h incubation under anaerobic conditions, focal strain density and total biomass were measured via colony counting and flow cytometry, and community composition and diversity were analysed via 16S rRNA sequencing.

In the absence of either antibiotic, focal strain density after 24 h was significantly lower in the presence of the three donor communities, indicative of strong competitive suppression (Fig. 1a). Surprisingly, we detected similarly strong competitive suppression in both the antibiotic treatments as we did in the antibiotic-free treatment. Instead, we found that focal strain performance was a stronger function of the specific donor community, irrespective of antibiotic treatment (Figs. 1b, and S2).

**Fig. 1:**
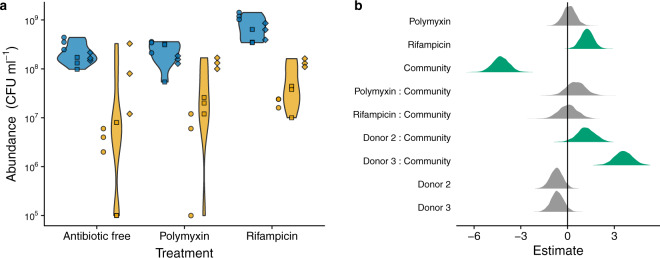
Effect of community, donor and antibiotic on focal strain abundance. **a** Violin plots showing the distribution of observed abundances of the focal strain in each antibiotic treatment. Blue denotes community free treatments; yellow denotes community treatment. Point shape denotes the individual human donor of live community or sterilized slurry: donor 1 = circles, donor 2 = squares, donor 3 = diamonds. **b** Treatment contrasts (posterior distributions of parameter estimates for a linear model with negative binomial errors) for focal strain abundance as a function of community (live vs sterile slurry), antibiotic (none, polymixin B or rifampicin), and donor (slurry prepared with samples from human donor 1, 2 or 3), and the interactions between community and antibiotic, and community and donor. Posterior parameter estimates in green have 95% credible intervals that do not overlap with 0 (i.e., there is less than 5% probability there is no effect of the variables/interactions captured by these coefficients). The reference level (vertical black line) = donor 1 in the no antibiotic treatment in the absence of the community (i.e., sterilized slurry).

What makes these results particularly striking is that, consistent with previous studies [7, 10, 13], treatment with a broad-spectrum antibiotic was still associated with a marked shift in community composition (analysis of 16S amplicon data) (Fig. 2a). Based on OTU composition, all three donors in the rifampicin treatment cluster separately from the polymyxin B and antibiotic-free treatments, which cluster together (Fig. 2b). This divergence in composition appears to be largely driven by enrichment of both Enterobacteriaceae and Erysipelotrichaceae in the rifampicin treatment (Fig. 2a). In addition to strong shifts in composition, total bacterial abundance was significantly reduced in the rifampicin treatment (Figs. 2c and S3). Despite this, total richness and diversity (Shannon Index) after 24 h did not differ between the treatments (Fig. 2c). In contrast, diversity loss over time was more strongly associated with donor identity, with the donor community associated with the weakest competitive suppression (donor 3) also exhibiting the largest decline in richness and diversity across all treatments. This observation is consistent with previous work demonstrating that colonization resistance in the mouse gut is highly contingent on the complexity and composition of the resident microbiota [14].

**Fig. 2:**
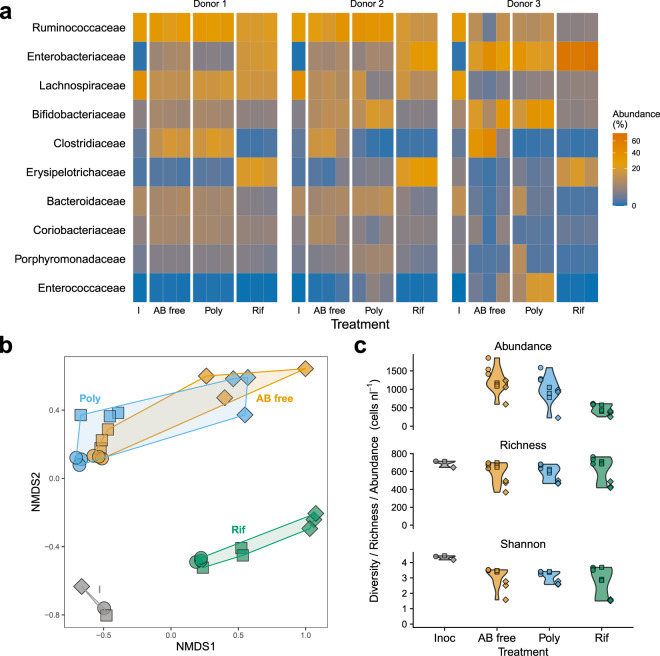
Community response to antibiotic treatments. **a** Heatmap of relative abundance of the ten most abundant families of bacteria across treatments (derived from amplicon data). I = inoculum; AB free = Antibiotic free; Poly = polymyxin B; Rif = rifampicin. **b** NMDS ordination of family level composition in each treatment-donor combination. **c** Violin plots showing the abundance (top), species richness (middle) and diversity (Shannon Index) (bottom) distributions in each treatment. In **b** and **c**: circles = donor 1; squares = donor 2, diamonds = donor 3.

A limitation of this study is that we only considered the effects of two antibiotics. Nevertheless, given the scale of community perturbation observed (Fig. 2), we can at least be sure our findings are not explained by a lack of antibiotic effects in our system. There must be some limit dictated by antibiotic concentration, combination, or duration of exposure, beyond which we would expect to observe stronger competitive release. Indeed, prior research has shown that antibiotics can greatly inhibit colonisation resistance [15, 16]. As such, characterizing where this limit lies (e.g., by investigating community-mediated suppression as a function of antibiotic concentration/duration) will be an important challenge for future work. Similarly, although we only considered a single focal strain, and other strains/species may have been more invasive (for example, those with fewer, different or less costly resistance mutations), key for our experiment was that the focal strain had a positive growth rate over the timescale of the experiment, despite exhibiting significant resistance costs in antibiotic-free assays (Fig. S1). This allowed us to test for sensitivity of competitive suppression to antibiotic treatment. We also note that in spite of a small boost in the focal strain’s performance in the presence of rifampicin independent of the community (a possible hormetic response [17] absent under aerobic growth in LB, Fig S1), we did not observe an increase in the magnitude of competitive release in the rifampicin treatment. Finally, the drop in diversity indicates, unsurprisingly, microcosms are a novel environment relative to the source environment. Despite this, key taxa in each community were stable over the course of the experiment, and previously over a longer timescale in the same set-up [9], demonstrating these conditions sustain diverse human-associated communities over relevant timescales.

In conclusion, these results are consistent with prevailing wisdom that healthy gut communities can suppress invading strains and thereby reduce the likelihood of resistance emerging [8, 9, 18]. Nevertheless, the absence of a significant effect of broad, or even narrow, spectrum antibiotics on the degree of competitive suppression of our focal strain is much more surprising. Despite the limitations of scope discussed above, this shows that the functional diversity of gut communities may be more robust to disturbance by broad spectrum antibiotics than previously recognised. This is not to suggest that the use of broad-spectrum antibiotics does not drive marked changes in composition but rather that there is some degree of functional redundancy in diverse communities that facilitates the maintenance of competitive suppression [12, 19]. Notwithstanding the need to test how these findings translate to in vivo settings, this finding is relevant for optimizing personalised treatments that either account for disruption by antibiotics or that make microbiomes harder for pathogens to invade.

## Supplementary information

Supplementary Information

## Supplementary information

The online version contains supplementary material available at 10.1038/s41396-021-00929-7. The datasets generated and analysed during the current study are available through the Dryad repository, 10.5061/dryad.d51c5b02r.
